# Sociodemographic characteristics and health-related quality of life of individuals undergoing antidepressant therapy

**DOI:** 10.1038/s41598-022-22164-6

**Published:** 2022-10-20

**Authors:** Abdullah A. Alfaifi, Abdullah U. Althemery

**Affiliations:** Department of Clinical Pharmacy, College of Pharmacy, Prince Sattam Bin Abdulaziz University, Al-Kharj, 11942 Saudi Arabia

**Keywords:** Quality of life, Outcomes research

## Abstract

An important factor for averting depression and creating awareness about clinical treatment is patient preference. Therefore, investigating health-related quality of life associated with different antidepressants is necessary. A retrospective cohort study was performed using the 2018 Medical Expenditure Panel Survey. The MEPS is a nationally representative database of the civilian and noninstitutionalized population spanning different ages, both sexes, and a wide range of sociodemographic and economic backgrounds. Differences in clinical and sociodemographic characteristics among patients using different antidepressant classes were explored. The differences in Veterans RAND 12-Item Health Survey (VR-12) results among groups were examined. The VR-12 metric was used since it measures a patient’s overall perspective of their health. Approximately 34.6 million of the patients reported using at least one antidepressant during 2018. Most patients receiving tricyclic therapy reported substantially better mental HRQoL than patients receiving selective serotonin reuptake inhibitors (SSRIs), serotonin-norepinephrine reuptake inhibitors (SNRIs), or combination therapy. Patients receiving atypical antidepressants reported substantially better mental HRQoL than those receiving other types of antidepressants. Most patients reported a substantial decline in HRQoL after SNRIs or combination therapy. This study found that HRQoL varied across antidepressant users. Thus, health care providers could benefit from taking into consideration quality of life when prescribing antidepressant agents. Moreover, further research is needed to explore other factors that could contribute to the quality of care for patients with depression.

## Introduction

Depression is a polygenic, highly complex, and common mental illness that remains a major burden on society^1,2^. It is a leading cause of disability worldwide and a major contributor to the global disease burden^3^. Undiagnosed and untreated depression results in a higher health burden and economic loss^4,5^. Although the effect of depression on cardiometabolic health is not immediately observed^6^, it affects both the physical and mental health of patients^7–9^. The physical health risks of individuals with depressive disorders are affected by the types of antidepressants administered to treat them^7^. Therefore, further research is needed to establish the population burden of antidepressants, particularly in a nationally representative database.

Several studies have been conducted to determine the effect of antidepressants on health-related quality of life (HRQoL). One study found a more favorable HRQoL effect among patients receiving SSRIs than among those given other antidepressants^10^. A comparative cohort study was conducted for patients who had depression vs. healthy subjects to examine HRQoL. The study concluded that the use of antidepressant therapy did not continue to improve patients’ HRQoL over time^11^. Another study argued that antidepressants are effective. Antidepressants have a positive impact on patients’ subjective and functional outcomes, such as disability, work functioning, social functioning, and well-being^12^. The differences in the clinical outcome results in these studies necessitate a study that compares antidepressant classes to shed some light on improving the care provided to these patients.

Effective treatments for depressive disorders have been reported^13^. However, in 2004, a study showed that patients delayed approaching a health care provider by almost a decade and that only 80% of patients with mental illness eventually received treatment^14^. In 2019, the number of adult patients receiving treatment for mental illness was even lower, at only 43.8%^15^. Accurate treatment for patients with mental illness can enhance their physical and mental status and encourage more individuals suffering from mental illness to receive treatment. One factor for averting depression and creating awareness about its clinical treatment is patient preference^16^. Therefore, it is necessary to investigate health-related quality of life (HRQoL) associated with different types of antidepressants. In addition to examining the sociodemographic characteristics of antidepressant users, investigating HRQoL associated with different types of antidepressants is imperative for exploring patients’ views regarding medication. Although the effects of clozapine and other SSRIs on general quality of life have been reported, the present study is the first to report HRQoL among antidepressant users^17,18^. This study examines sociodemographic and clinical characteristics among antidepressant users. Moreover, it explores HRQoL for patients receiving antidepressant medications.

## Results

### Sociodemographic and clinical characteristics

**Figure 1:**
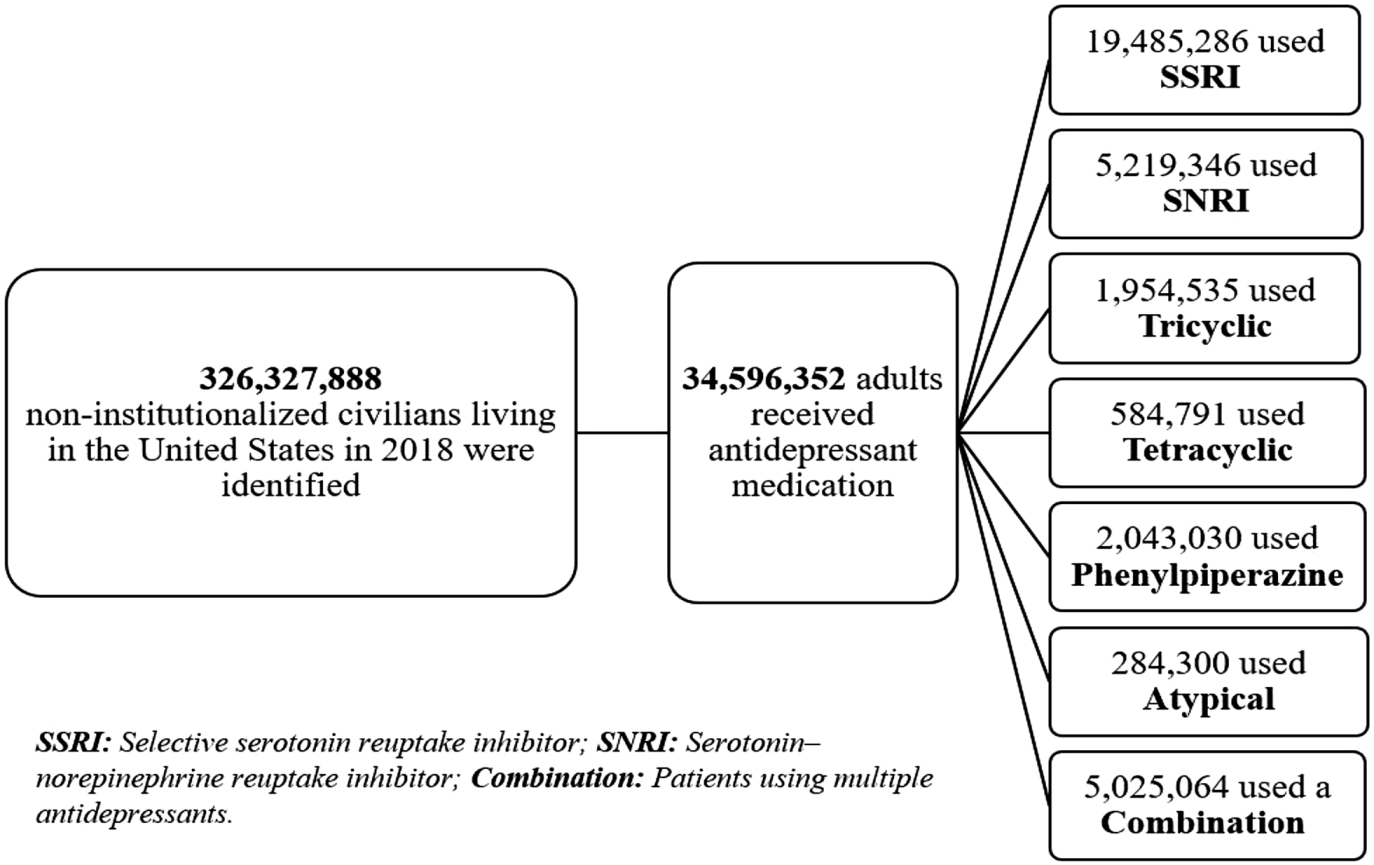
Flowchart of patients using antidepressant therapy in the MEPS.

Approximately 10.6% (34.6 million) of the U.S. civilian population was found to have received at least one antidepressant prescription in 2018 (Fig. 1).

Sociodemographic and comorbid variables and their cross-tabulated frequencies and statistics are presented in Table 1. Most antidepressant users were females. Patients who identified as Caucasian in the race/ethnicity item predominated in all groups, whereas Hispanic individuals accounted for 12.3% of patients who used atypical antidepressants. The proportion of African American patients receiving drugs was almost the same across all drug groups; however, the number of African American patients using tetracyclic antidepressants was 10% higher than that using other drugs. The proportion of patients on tetracyclic antidepressants who had poor or near-poor socioeconomic status was higher than that of other patients. There were significant differences in the percentage of patients using antidepressants based on family income. In total, 56.3% of patients using tricyclic antidepressants were married. In contrast, among patients who had never been married, SSRI use was more common than the use of other antidepressants. A significant difference was observed in the marital status of patients using different antidepressant types.

**Table 1:** Percentages of sociodemographic and clinical characteristics among antidepressant users.

	SSRIs N = 19,485,286	SNRIs N = 5,219,346	Tricyclic N = 1,954,535	Tetracyclic N = 584,791	Phenylpiperazine N = 2,043,030	Atypical N = 284,300	Combination N = 5,025,064
**Characteristics**							
	%	%	%	%	%	%	%
Age***							
18–44	33.1	24.2	17.9	14.1	25.5	44.9	21.6
45–64	34.4	42.5	46.7	27.4	33.1	37.4	44.3
65 +	32.5	33.3	35.4	58.5	41.4	17.7	34.1
Sex***							
Male	31.7	28.7	36.7	50.3	43.7	39.3	27.7
Female	68.3	71.3	63.3	49.7	56.3	60.7	72.3
Race/Ethnicity**							
Hispanic	9.6	6.2	10.0	7.6	10.2	12.3	5.5
Caucasian	80.5	82.9	76.6	73.6	76.9	77.3	81.2
African American	4.3	6.6	7.4	16.5	8.5	5.4	7.0
Other race/ethnicity	5.6	4.3	6.0	2.3	4.4	5.0	6.3
Marital status*							
Married	46.9	51.1	56.3	33.6	44.8	45.7	41.0
Previously married	27.6	33.1	29.6	44.8	37.7	30.6	36.1
Never married	25.5	15.8	14.1	21.6	17.5	23.7	22.9
Family income**							
Poor/low income	27.6	30.8	32.8	48.3	41.6	28.9	41.1
Middle income	30.0	27.7	29.2	23.7	22.6	15.9	27.0
High income	42.4	41.5	38.0	28.0	35.8	55.2	31.9
Educational level***							
12th grade or below	14.6	11.2	24.2	26.7	17.2	16.1	16.5
High school graduate	40.4	46.4	30.0	39.0	47.4	30.4	42.8
Some college/college graduate	45.0	42.4	45.8	34.3	35.4	53.5	40.7
Smoking**							
Yes	17.4	18.8	23.9	24.2	28.5	12.5	25.5
No	82.6	81.2	76.1	75.8	71.5	87.5	74.5
Comorbid conditions*							
2 +	40.1	58.2	56.4	50.4	50.7	45.5	52.2
1	26.9	22.0	25.8	33.7	23.8	10.3	28.9
0	33.0	19.8	17.8	15.9	25.5	44.2	18.9

SSRIs: Selective serotonin reuptake inhibitors; SNRIs: Serotonin and norepinephrine reuptake inhibitors; Combination: Patients using multiple antidepressants.

* *p* < 0.05; ** *p* < 0.01; *** *p* < 0.001.

The education level of the vast majority of surveyed patients was high school or above. The proportion of phenylpiperazine users who were smokers was the highest (0.28), whereas the proportion of atypical users who were smokers was the lowest (0.14). The difference in these proportions was significant. The frequency of comorbid conditions was higher among patients using SNRIs and tricyclic antidepressants than among the remaining patients (Table 1). A chi-square test was performed to identify any difference in comorbid conditions among different antidepressant groups, and a significant difference in comorbid conditions was found among the groups.

### HRQoL of antidepressant agent users

The medians and ranges of mental HRQoL for patients using antidepressants are presented in Table 2. Tricyclic antidepressant users exhibited the highest mental component summary score. Atypical antidepressants showed a more favorable effect than SNRIs and tetracyclic antidepressants. Patients on multiple antidepressants showed disparate outcomes in terms of mental HRQoL. The Kruskal–Wallis test was performed to examine differences in mental HRQoL between the different antidepressant groups; there was a significant difference in the outcomes (χ2 = 61.18, *p* < 0.0001, df = 6). The Dwass–Steel–Critchlow–Fligner test for multiple comparisons revealed that tricyclic antidepressants significantly enhanced mental HRQoL compared with SSRIs, SNRIs, and combination therapy (Table 3). In addition, phenylpiperazine and SSRIs alone were significantly more effective in improving mental HRQoL than combination antidepressants.

**Table 2:** Mental health-related quality of life associated with antidepressants***.

Types of antidepressants	Median	95% confidence intervals	
		Distribution free	
Phenylpiperazine	47.9	45.8	51.5
SNRIs	46.8	44.8	48.5
SSRIs	47.8	47.0	48.6
Tricyclic	51.6	48.7	54.3
Tetracyclic	47.1	41.4	51.4
Atypical	48.4	34.7	52.8
Combination	41.1	40.1	43.1

SSRIs: Selective serotonin reuptake inhibitors; SNRIs: Serotonin and norepinephrine reuptake inhibitors; Combination: Patients using multiple antidepressants.

***p < 0.001.

**Table 3:** Pairwise comparisons of antidepressants on mental HRQoL using the Dwass–Steel–Critchlow–Fligner test.

Comparison*	D-S-C-FW statistic	Median difference	*p* value
Tricyclic versus Combination	− 6.53	10.5	< 0.0001
Phenylpiperazine versus Combination	− 4.46	6.7	0.0002
SSRIs versus Combination	− 6.48	6.7	< 0.0001
Tricyclic versus SNRIs	− 3.65	4.8	0.0048
Tricyclic versus SSRIs	− 3.48	3.8	0.0088

SSRIs: Selective serotonin reuptake inhibitors; SNRIs: Serotonin and norepinephrine reuptake inhibitors; Combination: Patients using multiple antidepressants.

*Only significantly different results are listed.

Physical HRQoL was also higher among patients using atypical antidepressants. Similar findings were observed for SSRIs (Table 4). A decline in physical HRQoL was observed among patients using phenylpiperazine, SNRIs, and tetracyclic antidepressants. However, patients who were on combination antidepressant therapy reported the worst physical HRQoL. The Kruskal–Wallis test revealed that there was a significant difference in the medians of physical HRQoL (χ^2^ = 116.15, *p* < 0.0001). The Dwass–Steel–Critchlow–Fligner test for multiple comparison analysis of median values of physical HRQoL showed that atypical antidepressants were significantly superior to other types of antidepressants, except for SSRIs, in enhancing physical HRQoL (Table 5). SSRIs were also effective in enhancing patients’ physical HRQoL compared with combination, tricyclic, and serotonin and norepinephrine reuptake inhibitor therapy. Indeed, SSRIs were better than atypical antidepressants when they were both compared with SNRIs. The sensitivity and multivariate analyses yielded the same results as the main analysis after controlling for sociodemographic and clinical variables. The results of the sensitivity and multivariate analyses are presented in Supplementary material 1.

**Table 4:** Physical health-related quality of life associated with antidepressant agents.

Types of antidepressants***	Median	95% confidence intervals	
		Distribution free	
Phenylpiperazine	43.2	38.9	45.1
SNRIs	41.3	38.7	44.5
SSRIs	48.4	47.5	49.5
Tricyclic	40.9	38.2	45.2
Tetracyclic	43.1	37.7	49.9
Atypical	53.5	48.3	55.6
Combination	39.2	36.9	41.3

SSRIs: Selective serotonin reuptake inhibitors; SNRIs: Serotonin and norepinephrine reuptake inhibitors; Combination: Patients using multiple antidepressants.

***Statistically significant at *p* < 0.001.

**Table 5:** Pairwise comparisons of antidepressant treatments on physical HRQoL using Dwass–Steel–Critchlow–Fligner statistics.

Comparison*	D-S-C-FW statistic	Median difference	*p* value
Atypical versus Combination	− 3.64	14.3	0.0050
Atypical versus Tricyclic	3.28	12.6	0.0175
Atypical versus Phenylpiperazine	3.09	10.3	0.0322
SSRIs versus Combination	− 8.44	9.2	< 0.0001
SSRIs versus Tricyclic	4.99	7.5	< 0.0001
SSRIs versus SNRIs	− 6.42	7.1	< 0.0001
SSRIs versus Phenylpiperazine	− 4.36	5.2	0.0003
Atypical versus SNRIs	3.14	5.1	0.0274

*Only significantly different results are listed.

SSRIs: Selective serotonin reuptake inhibitors; SNRIs: Serotonin and norepinephrine reuptake inhibitors; Combination: Patients using multiple antidepressants.

## Discussion

In the present study, we aimed to analyze the HRQoL of patients receiving antidepressants, considering the differences in sociodemographic and clinical characteristics, and investigate the association of HRQoL with different classes of antidepressants. We found that antidepressant use was more prevalent among women than men, which was consistent with the findings of other studies^19,20^. Studies have shown that men seek medical care for depression more often than women^20^. However, antidepressant therapy is prescribed to men to a lesser extent, which is a sign of undertreatment. Moreover, health care providers tend to prescribe antidepressants to women more often than men without reporting a diagnosis of depression^21^. The higher use of antidepressants by women than by men might also be due to biological factors, as women experience lower serotonin levels, along with hormonal changes and sociocultural factors, which play important roles in the development of mental health conditions^22^. However, the current study examined antidepressant use solely and overlooked other types of treatments such as cognitive-behavioral therapy. Consequently, the results should be interpreted carefully.

This study showed that tetracyclic antidepressants were among the least used therapies, even with their favorable properties. Tetracyclic antidepressants are the most selective noradrenaline uptake inhibitors among the cyclic antidepressants currently used^23^. Tetracyclic antidepressants have moderate antihistaminic properties and few anticholinergic side effects^24^.

More than 80% of patients on combination therapy had at least one comorbid condition and were above the age of 45 years. Aging people with mental disorders are more likely to have polypharmacy than their counterparts^25^. The present study examined HRQoL changes in a large cohort of patients using different antidepressants. HRQoL improved in most patients using atypical or SSRI antidepressants. None of the surveyed patients reported the use of any other type of antidepressant therapy, such as monoamine oxidase inhibitors or unicyclic antidepressants. Tricyclic antidepressants yielded a better mental HRQoL and showed an advantage over well-known and frequently used antidepressants, such as SSRIs. The outstanding performance of tricyclic therapy, however, was not enough to increase the frequency of its use. It has been established that patients with depression are less motivated to overcome the odds to achieve successful antidepressant therapy. In addition, side effects or other requirements for treatment may lead to nonadherence^26^. Moreover, tricyclic therapy leads to poor physical HRQoL, which might be due to its cardiovascular effects and low tolerance. SSRIs confer reasonably better effects on both physical and mental health. Nonetheless, atypical antidepressants were best at enhancing patient HRQoL. Atypical antidepressants have several sites of action; however, they do not affect the sites known to elicit side effects or tolerance^27^. Patients on combination therapy had the worst physical and mental HRQoL. An association between the administration of combination medication and treatment resistance has been reported^28^. This indicates that most patients engage in combination therapy because they develop resistance to the first antidepressant therapy used, which may explain the worse mental HRQoL. Furthermore, the use of several medications means that patients are at an increased risk for medication-related adverse events, which may explain the poor physical HRQoL of these patients.

Although this study contributes to the literature, it was not without limitations. The MEPS database provided valuable information on a wide range of sociodemographic and economic sectors within the U.S. noninstitutionalized civilian population. Nevertheless, the MEPS data collection approach has several drawbacks. Data on previous treatment failure, medical history of participants, and antidepressant therapy contraindications are lacking. Researchers can expect a degree of underreporting for chronic conditions. Indeed, it has been shown that MEPS data underrepresent some mental diseases^29^. To help mitigate the effect of this limitation, this investigation focused on comparing HRQoL associated with different antidepressants rather than producing national estimates for depression.

Future studies comparing treatment groups using different parameters are needed. Indeed, other indirect economic results and disease-specific quality of life measurements should be studied to explore the effect of different antidepressants on patients with mental illnesses. This study is beneficial for patients, health care providers, policy-makers, legislators, payers, and pharmaceutical managers. Studies pertaining to research on patients suffering from depression are lacking. The results of this study highlighted the socioeconomic barriers that patients with depression face nationwide when seeking therapy. Health care practitioners must consider all factors to implement effective interventions for the more vulnerable group of patients who show an increased susceptibility to experiencing unfavorable outcomes. The present study provides an unbiased overview and includes all categories of therapy. Practitioners prescribing antidepressants should also use a similar perspective when determining treatment.

## Conclusions

The results confirmed the greater use of antidepressant therapy among women, married patients, and patients with two or more comorbid conditions than their counterparts. In addition, the results showed that patients using SNRIs and combination therapy experienced a decline in both physical and mental HRQoL. The findings of the present study can benefit health care providers by encouraging them to take the type of antidepressant into account as a factor that affects depression management. Additionally, this study can help patients and health care providers choose the appropriate treatment options and best medication regimens.

## Material and methods

### Study population and independent variables

The Medical Expenditure Panel Survey (MEPS), a nationally representative survey of the U.S. civilian noninstitutionalized population, was selected as the data source for this study. The MEPS is a collection of surveys intended to provide estimates for payment source, health status, health insurance coverage, and health care utilization and expenditure^30^. However, those who live in an institution (e.g., nursing home or prison), were in the military, or lived out of the country were not included in the MEPS. The subjects were adults aged 18 years or older who received any class of antidepressant according to the Multum Lexicon therapeutic classification system^31^. Data were extracted from the MEPS database between January 1, 2018, and December 31, 2018, for subjects who met the inclusion criteria.

The MEPS 2018 data and authorization forms follow the Health Insurance Portability and Accountability Act (HIPAA). The Westat Institutional Review Board reviewed the HIPAA compliance of the MEPS forms. The confidentiality of the MEPS data is addressed in Sect. 924(c) of the Act^32^. All participants provided informed consent prior to their participation in the MEPS. All experimental protocols were approved by Westat, Inc and Research Triangle Institute under the authority of the Public Health Research Act.

Each event in the MEPS Prescribed Medicines file, which represents a unique medication, provides complete identification information for each medication. Two of these variables are therapeutic class (TC1) and therapeutic subclass (TC1S1), which are adapted from Cerner Multum, Inc.’s Multum Lexicon variables^31^. The main variable of interest was the type of antidepressant agent, which was considered a categorical variable. This variable was classified into seven categories: SSRIs, tricyclic antidepressants, atypical antidepressants, phenylpiperazine antidepressants, tetracyclic antidepressants, SNRIs, and combination therapy of multiple antidepressant treatments. The combination therapy class was defined as patients who were on more than one antidepressant therapy during the survey year.

Sociodemographic and clinical variables were considered before assessing the association between the use of antidepressants and HRQoL outcomes. The sociodemographic variables were recorded on a self-reported basis by administering a paper-and-pencil questionnaire during MEPS visits. The variables selected for this study were age, sex, race/ethnicity, educational attainment, marital status, family income, smoking status, and insurance status. Comorbid conditions were defined as medical conditions that have been specified by the Agency for Healthcare Research and Quality as priority conditions. These conditions were hypertension, heart disease, high cholesterol, emphysema, chronic bronchitis, diabetes, cancer, arthritis, asthma, attention-deficit/hyperactivity disorder, and stroke.

### Study outcome

The outcome of this study was HRQoL. HRQoL has been recognized as a measure of QoL specifically related to health and reflects the perceived physical and mental health of an individual^33^. The Veterans RAND 12-Item Health Survey is a self-administered health survey comprising 12 items to measure HRQoL, estimate disease burden, and evaluate the disease-specific impact on general and selected populations^34^. The VR-12 is a paper-and-pencil questionnaire that was fielded during Panel 21 Round 4 and Panel 22 Round 2 of the MEPS and collected in 2018. The survey was designed to collect a variety of health status and health care quality measures of adults aged 18 and older. The following 12 items were included in the questionnaire: general health, limitations in moderate activities or climbing several flights of stairs, accomplished or worked less than usual due to physical issues, accomplished or worked less than usual due to emotional issues, pain interference in life, general state of feeling (calm, energetic, or blue), and experience of emotional and physical issues during social activities. The 12 items in the questionnaire correspond to two principal physical and mental HRQoL domains, with higher scores denoting better health status.

### Data analysis

Patients receiving different types of antidepressants were compared using the chi-square test for categorical sociodemographic and clinical variables. Differences between the physical and mental component summaries and types of antidepressants were analyzed using the Kruskal–Wallis test. The Kruskal–Wallis test was performed because at least one class of antidepressants did not follow a normal distribution. The post hoc Dwass–Steel–Critchlow–Fligner multiple comparison test was performed to determine the groups that were significantly different. This post hoc test is a two-sided test that accounts for type I error. The level of significance for all statistical tests was set at *p* < 0.05. All analyses accounted for MEPS sampling weights and balanced repeated replication methods for the stratified and clustered survey design. The R (v3.5.3) Studio-integrated development environment was used to perform statistical analysis. All methods were carried out in accordance with relevant guidelines and regulations. Sensitivity analysis was conducted assuming that HRQoL was normally distributed. Moreover, a regression model was established with HRQoL as the outcome variable and the type of antidepressant treatment as the independent variable controlling for sociodemographic and clinical characteristics (age, sex, race/ethnicity, educational attainment, marital status, family income, smoking status, insurance status and comorbid conditions).

## Supplementary Information

Supplementary Information.

## Data Availability

The data used in this study are available in modern Microsoft Excel spreadsheet format from the corresponding author to any researcher/scientist upon request. The MEPS data supporting the conclusion of the article are also available publicly at the Agency for Healthcare Research and Quality, [https://www.meps.ahrq.gov/mepsweb/index.jsp].

## References

[1] The National Institute of Mental Health. *Depression basics* (The National Institute of Mental Health, 2020).

[2] Dunn EC (2015). Genetic determinants of depression: Recent findings and future directions. Harv. Rev. Psychiatry.

[3] GBD 2017 Disease and Injury Incidence and Prevalence Collaborators. Global, regional, and national incidence, prevalence, and years lived with disability for 354 diseases and injuries for 195 countries and territories, 1990–2017: A systematic analysis for the Global Burden of Disease Study 2017. *Lancet* **392,** 1789–1858 (2018). 10.1016/S0140-6736(18)32279-7 PMC6227754 30496104

[4] Thilina D, Yadurshini R (2020). The economic burden of depression: Why we should Invest in treatment and prevention. Int. J. Clin. Stud. Med. Case Rep..

[5] Luppa M, Heinrich S, Angermeyer MC, König HH, Riedel-Heller SG (2007). Cost-of-illness studies of depression: A systematic review. J. Affect. Disord..

[6] Prince M (2007). No health without mental health. Lancet.

[7] Firth J (2019). The Lancet Psychiatry Commission: A blueprint for protecting physical health in people with mental illness. Lancet Psychiatry.

[8] Vancampfort D (2015). Risk of metabolic syndrome and its components in people with schizophrenia and related psychotic disorders, bipolar disorder and major depressive disorder: A systematic review and meta-analysis. World Psychiatry.

[9] Vancampfort D (2016). The prevalence of metabolic syndrome in alcohol use disorders: A systematic review and meta-analysis. Alcohol Alcohol.

[10] Taylor AT, Spruill WJ, Longe RL, Wade WE, Wagner PJ (2001). Improved health-related quality of life with SSRIs and other antidepressants. Pharmacotherapy.

[11] Almohammed OA (2022). Antidepressants and health-related quality of life (HRQoL) for patients with depression: Analysis of the medical expenditure panel survey from the United States. PLoS ONE.

[12] Andrade C (2022). Antidepressant drugs and health-related quality of life: A reader’s guide on how to examine a “viral” research paper with a critical eye. J. Clin. Psychiatry.

[13] Wang PS (2007). Use of mental health services for anxiety, mood, and substance disorders in 17 countries in the WHO world mental health surveys. Lancet.

[14] Wang PS, Berglund PA, Olfson M, Kessler RC (2004). Delays in initial treatment contact after first onset of a mental disorder. Health Serv. Res..

[15] Lipari, R. N. & Park-Lee, E. *Key Substance Use and Mental Health Indicators in the United States: Results from the 2018 National Survey on Drug Use and Health* (SAMHSA, 2020).

[16] Solmi M (2021). How can we improve antidepressant adherence in the management of depression? A targeted review and 10 clinical recommendations. Braz. J. Psychiatry.

[17] Paton C, Anderson IM, Cowen PJ, Delgado O, Barnes TRE (2020). Prescribing for moderate or severe unipolar depression in patients under the long-term care of UK adult mental health services. Ther. Adv. Psychopharmacol..

[18] Maher S (2016). Clozapine-induced hypersalivation: An estimate of prevalence, severity and impact on quality of life. Ther. Adv. Psychopharmacol..

[19] Noordam R (2015). Prescription and indication trends of antidepressant drugs in the Netherlands between 1996 and 2012: A dynamic population-based study. Eur. J. Clin. Pharmacol..

[20] Boyd A (2015). Gender differences in psychotropic use across Europe: Results from a large cross-sectional, population-based study. Eur. Psychiatry.

[21] Sundbom LT, Bingefors K, Hedborg K, Isacson D (2017). Are men under-treated and women over-treated with antidepressants? Findings from a cross-sectional survey in Sweden. BJPsych Bull..

[22] Dennerstein L, Soares CN (2008). The unique challenges of managing depression in mid-life women. World Psychiatry.

[23] Cowen PJ, Bellack AS, Hersen M (1998). Psychopharmacology. Comprehensive Clinical Psychology.

[24] Marano G (2011). Cardiologic side effects of psychotropic drugs. J. Geriatr. Cardiol..

[25] de Lima JD, Teixeira IA, Silva FO, Deslandes AC (2020). The comorbidity conditions and polypharmacy in elderly patients with mental illness in a middle income country: A cross-sectional study. IBRO Rep..

[26] Gautam S, Jain A, Gautam M, Vahia VN, Grover S (2017). Clinical practice guidelines for the management of depression. Indian J. Psychiatry.

[27] Horst WD, Preskorn SH (1998). Mechanisms of action and clinical characteristics of three atypical antidepressants: Venlafaxine, nefazodone, bupropion. J. Affect. Disord..

[28] Dold M (2018). Clinical correlates of augmentation/combination treatment strategies in major depressive disorder. Acta Psychiatr. Scand..

[29] Slade EP, Goldman HH, Dixon LB, Gibbons B, Stuart EA (2015). Assessing the representativeness of medical expenditure panel survey inpatient utilization data for individuals with psychiatric and nonpsychiatric conditions. Med. Care Res. Rev..

[30] Agency for Healthcare Research and Quality. *The Medical Expenditure Panel Survey* (Agency for Healthcare Research and Quality, 2020).

[31] Centers for Disease Control and Prevention. *National Health and Nutrition Examination Survey: 2013–2014 Data Documentation, Codebook, and Frequencies* (Centers for Disease Control and Prevention, 2020).

[32] Cohen, J. *Methodology Report #1: Design and Methods of the Medical Expenditure Panel Survey Household Component* (Agency for Health Care Policy and Research, 1997).

[33] Sitlinger A, Zafar SY (2018). Health-related quality of life: The impact on morbidity and mortality. Surg. Oncol. Clin. N. Am..

[34] Honeycutt, A. A., Hoerger, T., Hardee, A., Brown, L. & Smith, K. *An Assessment of the State of the Art for Measuring Burden of Illness: Final Report* (U.S. Department of Health and Human Services, 2011).

