# Supplementary material for: Sociodemographic characteristics and health-related quality of life of individuals undergoing antidepressant therapy
Source: Sci Rep. 2022 Oct 20;12:17518. doi: 10.1038/s41598-022-22164-6 (PMC9584901; doi:10.1038/s41598-022-22164-6)
Supplement: Supplementary file 1 — Supplementary Information. [file 41598_2022_22164_MOESM1_ESM.docx]

Sociodemographic Characteristics and Health-related Quality of Life of Individuals Undergoing Antidepressant Therapy

Abdullah A Alfaifi and Abdullah U Althemery

Supplementary Table S1. Physical health-related quality of life associated with antidepressants

| Treatment Type | Estimate | Std Error | t-Statistics | *p* value |
| --- | --- | --- | --- | --- |
| Combination | 40.90 | 0.73 | 56.19 | <.0001 |
| Atypical | 47.49 | 2.30 | 20.65 | <.0001 |
| Phenylpiperazine | 42.31 | 0.98 | 43.15 | <.0001 |
| SNRIs | 41.96 | 0.68 | 61.19 | <.0001 |
| SSRIs | 44.35 | 0.49 | 89.24 | <.0001 |
| Tricyclic | 42.63 | 1.02 | 41.71 | <.0001 |
| Tetracyclic | 46.49 | 1.75 | 26.47 | <.0001 |
| **R-square** | 0.3410 | Controlling for age, sex, race, marital status, income, education, smoking, and number of comorbid conditions | | |
| **Number of Observation** | 2,726 |  |  |  |
| **F value** | 78.94 |  |  |  |
| ***p* value** | <.0001 |  |  |  |

SSRIs: Selective serotonin reuptake inhibitors; SNRIs: Serotonin and norepinephrine reuptake inhibitors; Combination: Patients using multiple antidepressants

Supplementary Table S2. Mental health-related quality of life associated with antidepressants.

| Treatment Type | Estimate | Std Error | t-Statistics | *p* value |
| --- | --- | --- | --- | --- |
| Combination | 41.15 | 0.79 | 52.41 | <.0001 |
| Atypical | 42.83 | 3.19 | 13.40 | <.0001 |
| Phenylpiperazine | 45.35 | 0.89 | 50.90 | <.0001 |
| SNRIs | 43.93 | 0.75 | 58.38 | <.0001 |
| SSRIs | 44.74 | 0.51 | 88.61 | <.0001 |
| Tricyclic | 47.89 | 1.02 | 46.86 | <.0001 |
| Tetracyclic | 44.78 | 1.41 | 31.79 | <.0001 |
| **R-square** | 0.1296 | Controlling for age, sex, race, marital status, income, education, smoking, and number of comorbid conditions | | |
| **Observation** | 2,726 |  |  |  |
| **F value** | 20.43 |  |  |  |
| ***p* value** | <.0001 |  |  |  |

SSRIs: Selective serotonin reuptake inhibitors; SNRIs: Serotonin and norepinephrine reuptake inhibitors; Combination: Patients using multiple antidepressants
